# Human‐induced habitat fragmentation effects on connectivity, diversity, and population persistence of an endemic fish, *Percilia irwini*, in the Biobío River basin (Chile)

**DOI:** 10.1111/eva.12901

**Published:** 2019-12-09

**Authors:** Francisca Valenzuela‐Aguayo, Gregory R. McCracken, Aliro Manosalva, Evelyn Habit, Daniel E. Ruzzante

**Affiliations:** ^1^ Laboratorio de Ecología y Conservación de Peces Departamento de Sistemas Acuáticos Facultad de Ciencias Ambientales y Centro EULA Universidad de Concepción Concepción Chile; ^2^ Department of Biology Dalhousie University Halifax NS Canada

**Keywords:** connectivity, conservation, dams, endangered species, fragmentation, genetic diversity, microsatellite

## Abstract

An understanding of how genetic variability is distributed in space is fundamental for the conservation and maintenance of diversity in spatially fragmented and vulnerable populations. While fragmentation can occur from natural barriers, it can also be exacerbated by anthropogenic activities such as hydroelectric power plant development. Whatever the source, fragmentation can have significant ecological effects, including disruptions of migratory processes and gene flow among populations. In Chile, the Biobío River basin exhibits a high degree of habitat fragmentation due to the numerous hydroelectric power plants in operation, the number of which is expected to increase following new renewable energy use strategies. Here, we assessed the effects of different kinds of barriers on the genetic structure of the endemic freshwater fish *Percilia irwini*, knowledge that is critically needed to inform conservation strategies in light of current and anticipated further fragmentation initiatives in the system. We identified eight genetic units throughout the entire Biobío system with high effective sizes. A reduced effective size estimate was, however, observed in a single population located between two impassable barriers. Both natural waterfalls and human‐made dams were important drivers of population differentiation in this system; however, dams affect genetic diversity differentially depending on their mode of operation. Evidence of population extirpation was found in two river stretches limited by upstream and downstream dams. Significant gene flow in both directions was found among populations not separated by natural or anthropogenic barriers. Our results suggest a significant vulnerability of *P. irwini* populations to future dam development and demonstrate the importance of studying basin‐wide data sets with genetic metrics to understand the strength and direction of anthropogenic impacts on fish populations.

## 1 INTRODUCTION

Understanding the factors that affect spatial distribution of genetic diversity in freshwater systems is fundamental for the design of robust management and conservation strategies. Theory predicts that, under migration–drift equilibrium, headwater populations in freshwater systems will exhibit lower genetic diversity than downstream populations. Extrapolation of such modeling predictions to the wild is, however, complicated by the fact that natural systems are in general not expected to be under equilibrium (Raeymaekers et al., 2008; Ruzzante et al., 2019), even less so when they are affected by varying degrees of natural and human‐induced fragmentation.

Natural waterfalls lead to habitat fragmentation and promote diversification on evolutionary timescales (e.g., speciation; Dias, Cornu, Oberdorff, Lasso, & Tedesco, 2013; Fagan, 2002; Losos & Parent, 2009). When habitat fragmentation results from anthropogenic activities such as hydropower dams, changes in connectivity usually take place over relatively short time frames (Faulks, Gilligan, & Beheregaray, 2011). Irrespective of the source(s), however, fragmentation as a landscape‐scale process (Fahrig, 2003) is known to affect both functional connectivity (e.g., dispersal and gene flow between habitat patches) and structural connectivity (e.g., habitat types and distance between habitat patches) (Brooks, 2003; Goodwin, 2003). Furthermore, the impacts on both structural and functional connectivity resulting from human‐induced habitat fragmentation will generally be exacerbated when numerous habitat alteration factors act synergistically. For instance, when numerous hydroelectric power plants are established within any given drainage and the drainage is further impacted by other anthropogenic effects (e.g., invasive species and water diversion/channeling initiatives that subtract water from its regular course (Sheridan, 1995)), the ultimate effect on any given species' genetic diversity is likely to be higher than just the sum of individual effects.

Barrier impact, though generally a function of the physical characteristics of the barrier, will often include both upstream and downstream habitat changes such as changes in flow regime, sediment transport, and temperature (Liermann, Nilsson, Robertson, & Ng, 2012; Rosenberg et al., 1997). Such habitat type changes can often lead to changes in species abundances (Hanks & Hartman, 2018; Hu, Hua, Zhou, Wu, & Wu, 2015). Populations that remain on opposite sides of a barrier will likely lose genetic diversity and experience increases in structure. The loss of genetic diversity may lead to a decline in the populations' ability to adapt to changes in the local environment, ultimately leading to declines in population sizes and an increased risk of extirpation (Horreo et al., 2011; Morita, Morita, & Yamamoto, 2009; Neraas & Spruell, 2001; Nielsen, Hansen, & Loeschcke, 1997). Hydroelectric power development has been an important cause for the loss of connectivity, habitat fragmentation, and degradation in streams and rivers worldwide, yet it is anticipated that by 2050, hydroelectric power generation capacity will be doubled from current levels to 3.121 TWh (Hancock & Sovacool, 2018).

The present study was conducted in the Biobío River basin, the basin with the most exploitable hydroelectric potential in Chile (Ministerio de Energía, 2016). The basin harbors three main rivers exhibiting different levels of human intervention with overall eleven hydroelectric power plants currently in operation. Plants differ substantially in the mode of operation and in the extent and type of change they inflict on the habitat and water flow regime (see Section 2). The basin is home to 18 out of 45 native fish species found throughout Chile (Campos, Ruiz, & Gavilán, 1993; Habit, Dyer, & Vila, 2006). Here, we use *Percilia irwini* (Eigenmann, 1927), a species endemic to the basin (Arratia & Quezada‐Romegialli, 2019), as a model to examine the effects of fragmentation on genetic diversity. *Percilia irwini* is a small (length ≤ 90 mm) relatively short‐lived (4 years) benthopelagic fish usually found in shallow areas (<1 m) with rocky substrate and slow (<0.5 m/s) moving waters (Habit & Belk, 2007). At low flow rates (5–15 cm/s), it maintains a fixed position on the bottom but higher flow rates (25–35 cm/s) stimulate swimming behavior with individuals eventually taking refuge behind rocks in areas of low velocity and turbulence (García, Sobenes, Link, & Habit, 2012). The species is considered resident and nonmigratory. It is also considered endangered due to its restricted distribution and habitat loss (Habit & Belk, 2007; Ministerio de Medio Ambiente, 2019). Its reproductive biology and life history have not been studied in detail; the risk to its genetic integrity in the face of increasing fragmentation is largely unknown.

Our aim in the present study was to estimate the genetic diversity (28 microsatellite markers), differentiation, and gene flow among *P. irwini* populations inhabiting the Biobío River basin. The ultimate goal is to understand how anthropogenic and natural habitat fragmentation has influenced patterns of neutral genetic variability in *P. irwini*. We hypothesized that (a) the presence of anthropogenic barriers (hydropower dams) resulted in higher levels of population genetic structure compared to natural barriers. (b) The level of population structure depends on the type of hydropower plant, whether reservoir or run of the river (see Section 2), with reservoir plants limiting gene flow more than run‐of‐the‐river plants. We assessed gene flow and population structure as a function of the type (natural vs. anthropogenic) of barriers between sampling sites, the time elapsed since their construction (if anthropogenic in origin), and their mode of operation (hydropeaking vs. run of the river). We report on two river sections located between power plants where *P. irwini* appears to have been extirpated from. Our study provides insights on the vulnerability of a resident nonmigratory species in the face of increased habitat fragmentation. We discuss management implications of our findings providing guidance for stakeholders and government agencies.

## 2 MATERIALS AND METHODS

### 2.1 The study area and hydroelectric power plants

**Figure 1:**
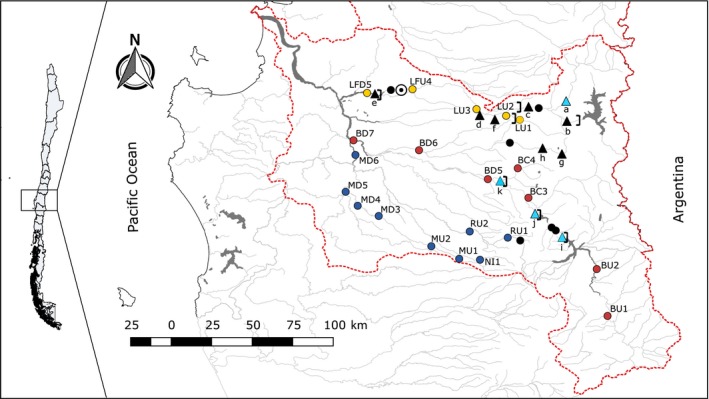
Biobío basin in central Chile, showing sampling sites and hydropower plants. Yellow circles: Laja River; Red circles: Biobío River; Blue circles: Renaico–Malleco River sample sites. Black circles: sample sites where *Percilia irwini* was absent. Black‐and‐white circle represents the “Salto del Laja” waterfall. Black triangles represent run‐of‐the‐river hydropower plants, and light blue triangles represent storage hydropower plants: (a) El Toro, (b) Abanico, (c) Antuco, (d) Quilleco, (e) Laja, (f) Rucúe, (g) Peuchén, (h) Mampil, (i) Ralco, (j) Pangue, and (k) Angostura. Black square brackets show the localization of the physical barriers

The Biobío River basin is currently home to 11 hydroelectric plants in operation in three main rivers from north to south geographically: Laja, Biobío, and Renaico–Malleco (Table 1, Figure 1). The oldest plant (Abanico) started operations in 1948 with the remaining 10 plants built beginning in the 1970s and up to the recent past as follows: El Toro (1973), Antuco (1981), Pangue (1996), Rucúe (1998), Peuchén (2000), Mampil (2000), Ralco (2004), Quilleco (2007), Angostura (2014), and Laja (2015) (Table 1). Four plants (El Toro, Pangue, Ralco, and Angostura) are “storage hydroelectric plants” which work through hydropeaking, a process characterized by rapid fluctuations in flow regime, depending on the demand of energy production (Bruder et al., 2016; Hauer, Holzapfel, Leitner, & Graf, 2017; Zimmerman, Letcher, Nislow, Lutz, & Magilligan, 2010). One plant (El Toro) is located in the Laja River and uses the natural Laja Lake as a reservoir. The other three (Ralco, Pangue, and Angostura) are located in the main channel of the Biobío River and impound a total of 70 km of the river. The other seven (Abanico, Antuco, Rucúe, Mampil, Peuchén, Quilleco, and Laja) are run‐of‐the‐river hydroelectric plants, these plants use the flow of water from a canalized river, to produce electricity on a continuous basis, and the water is subsequently returned to the river without chemical or physical changes (Lazzaro, Basso, Schirmer, & Botter, 2013). Regardless of the type of operation, whether storage or run of the river, each power plant may or may not have a physical barrier fragmenting the river channel. In this study, “barrier” refers to a natural or anthropogenic wall. For natural walls, waterfalls >10 m high were considered barriers preventing upstream movement of fish. Dams >10 m in height were considered barriers preventing both upstream and downstream fish movement. None of the existing hydropower plants exhibit fish passes or other mitigation measures to improve connectivity. One more hydroelectric power plant has recently been approved and will be located directly downstream of Angostura in the Biobío River (Rucalhue; RCA No. 159, Servicio de Evaluación Ambiental, 2016). The basin also exhibits a natural waterfall, “Salto del Laja” (Figure 1).

**Table 1:** Characteristics of the hydroelectric power plants in the Biobío River system

River	Hydroelectric power plant	Type of power plant	Year	Power capacity (MW)	Physical barrier	Reservoir size (mill. m^3^)
Laja	Abanico	RoR	1948	136.0	Yes	na
	El Toro	Storage	1973	450.0	No	7.700 (natural Laja Lake used as reservoir)
	Antuco	RoR	1981	320.0	No	na
	Rucúe	RoR	1998	178.4	Yes	na
	Quilleco	RoR	2007	70.8	No	na
	Laja	RoR	2015	34.4	Yes	na
Biobío	Ralco	Storage	2004	690.0	Yes	1.222
	Pangue	Storage	1996	467.0	Yes	175
	Angostura	Storage	2014	323.8	Yes	100
	Mampil^a^	RoR	2000	55.0	No	na
	Peuchén^a^	RoR	2000	85.0	No	na

Note

Type of power plant indicates whether storage hydropower (with dam and reservoir) or run of the river (RoR; no reservoir and lateral water intake). Year: indicates year operations started. The presence or absence of a physical barrier and reservoir is given for each plant.

Abbreviation: na, not applicable.

^a^ Located in the Duqueco River, tributary of the Biobío.

### 2.2 Sampling design and collections

*Percilia irwini* were collected by seine netting or electrofishing, over two consecutive years (2016 and 2017) with 13 out of 21 sites (distributed among the three major rivers comprising the Biobío system) visited only once, either in 2016 or in 2017, and eight sites visited on both years to increase sample sizes (Table 2). A further six sites were visited, but five provided no *P. irwini* individuals and one provided only four fish and was not considered further. Fish (*N = *927) were measured (total length) and weighed. Fin clips were stored in 96% ethanol for subsequent DNA analysis. Fish were genotyped at a final total of 28 species‐specific microsatellites (Yu et al., 2018) developed and genotyped in the Marine Gene Probe Laboratory at Dalhousie University, Nova Scotia, Canada.

**Table 2:** Sampling site information for *Percilia irwini* from the Biobío River system, including sample ID, coordinates (Lat. and Long.), Elevation (masl), sample size, *N* (2016, 2017), allelic richness (*A*_R_), expected heterozygosity (*H*_e_), observed heterozygosity (*H*_o_), final population names (LU = Laja upstream, LD = Laja downstream, BU = Biobío upstream, BC = Biobío central, BD = Biobío downstream, BD_(outlet)_ = Biobío downstream outlet, RM = Renaico–Malleco, NI = Niblinto), and effective population size $\left({\hat{N}}_{\text{eLD}}\right)$ with and without migrants, including lower and upper limits. ∞ = infinite

Main rivers	Site	Affluent river	Location	Sample ID	Latitude (S)	Longitude (W)	Elevation (masl)	*N*	*A*_R_	*H*_e_	*H*_o_	Population name	*A*_R_ population	*H*_e_ population	*H*_o_ population	*N*_e_ $\left({\hat{N}}_{\text{eLD}}\right)$with migrants	*N*_e_ $\left({\hat{N}}_{\text{eLD}}\right)$without migrants
Laja	LU1	Laja	Laja upstream	BIO23	37°22′23.29	71°44′24.39	531	49, –	5.46	0.650	0.612						
	LU2	Laja	Laja upstream	BIO22	37°20′32.70	71°48′0.30	457	30, 10	5.36	0.645	0.610	LU	7.61	0.655	0.614	∞ (2,630–∞)	∞ (2,493–∞)
	LU3	Laja	Laja upstream	BIO25	37°19′18.78	71°57′54.56	312	20, –	5.32	0.626	0.620						
	LFU4	Laja	Laja fall upstream	BIO7	37°12′39.34	72°19′15.73	189	38, –	5.63	0.651	0.618						
	LFD5	Laja	Laja fall downstream	BIO9	37°13′55.15	72°34′25.46	108	54, –	6.47	0.697	0.679	LD	9.13	0.697	0.679	∞ (1,763–∞)	∞ (2,201–∞)
Biobío	BU1	Biobío	Biobío upstream	BIO1	38°28′23.60	71°14′10.14	895	21, 15	4.51	0.550	0.539	BU	5.73	0.567	0.544	∞ (849–∞)	∞ (849–∞)
	BU2	Biobío	Biobío upstream	BIO2	38°12′43.99	71°17′46.13	745	35, 16	4.46	0.571	0.547						
	BC3	Biobío	Biobío central	BIO5	37°48′55.56	71°40′34.72	342	45, 12	5.23	0.606	0.567	BC	7.60	0.606	0.568	935 (372–∞)	745 (322–∞)
	BC4	Biobío	Biobío central	BIO17	37°39′3.72	71°44′7.41	417	14, –	4.95	0.568	0.572						
	BD5	Biobío	Biobío downstream	BIO6	37°42′41.67	71°54′6.98	263	38, 44	6.13	0.654	0.623	BD	8.99	0.654	0.623	∞ (1,290–∞)	∞ (897–∞)
	BD6	Biobío	Biobío downstream	BIO21	37°33′2.63	72°17′5.06	135	42, –	6.45	0.673	0.617	BD_(outlet)_	9.39	0.672	0.621	1,048 (467–∞)	1,058 (475–∞)
	BD7	Biobío	Biobío downstream	BIO15	37°29′43.54	72°38′56.97	56	37, –	6.40	0.660	0.625						
Renaico–Malleco	RU1	Renaico	Renaico upstream	BIO18	38° 2′14.07	71°47′27.81	507	22, 14	6.46	0.662	0.599						
	RU2	Renaico	Renaico upstream	BIO28	38° 0′10.92	72° 0′9.78	306	–, 26	6.21	0.658	0.639						
	MU1	Malleco	Malleco upstream	BIO11	38° 9′19.06	72° 3′40.60	422	47, –	6.06	0.651	0.608						
	MU2	Malleco	Malleco upstream	BIO12	38° 5′6.33	72°12′58.68	283	49, –	6.14	0.648	0.6	RM	9.23	0.668	0.608	7,701 (2,579–∞)	9,169 (2,705–∞)
	MD3	Malleco	Malleco downstream	BIO27	37°55′0.41	72°30′32.04	139	–, 28	6.13	0.639	0.601						
	MD4	Malleco	Malleco downstream	BIO13	37°51′35.62	72°37′32.93	93	32, 40	6.29	0.668	0.625						
	MD5	Malleco	Malleco downstream	BIO26	37°46′55.76	72°41′34.23	69	–, 16	6.00	0.639	0.593						
	MD6	Malleco	Malleco downstream	BIO14	37°34′36.91	72°38′17.87	57	49, –	6.42	0.667	0.591						
	NI1	Niblinto	Niblinto upstream	BIO10	38°9′37.90	71°56′43.91	533	55, 29	5.30	0.629	0.569	NI	7.2	0.629	0.569	637 (337–∞)	729 (339–∞)

### 2.3 DNA extraction and microsatellite genotyping

Fin clips (5–10 mg per individual) were digested at 55°C for approximately 8 hr using proteinase *K* (Bio Basic Inc.). DNA was then extracted using a MultiPROBE II Plus Liquid Handling System (PerkinElmer) using a glass milk protocol modified from Elphinstone, Hinten, Anderson, and Nock (2003).

Identification and characterization of microsatellites were conducted as described in Yu et al. (2018). Microsatellites were scored with Megasat (Zhan et al., 2017) and were subsequently subject to a standard battery of analytical methods. Potential genotyping errors, presence of null alleles, and stuttering were assessed with MICRO‐CHECKER 2.2.3 (Van Oosterhout, Hutchinson, Wills, & Shipley, 2004). Arlequin 3.5.1.3 (Excoffier & Lischer, 2010) was used to test for linkage disequilibrium (LD) and Hardy–Weinberg equilibrium (HWE); these tests were conducted using 10,000 permutations and 1,000,000 Markov chain steps and 100,000 dememorization steps, respectively, with a false discovery rate of 5%. Outlier analyses were performed using the Bayesian program BayeScan 2.1 (Foll & Gaggiotti, 2008) to identify putative loci under selection. Out of the initial 33 microsatellites examined, five appeared to exhibit null alleles or departures from Hardy–Weinberg and were thus excluded from further analysis. We therefore conducted the present study with a battery of 28 microsatellite markers.

### 2.4 Estimating genetic diversity and population structure

We estimated allele frequencies and allelic richness (*A*_R_) per sample site using FSTAT 2.9.4 (Goudet, 2001). We estimated observed (*H*_o_) and expected heterozygosities (*H*_e_) for each sampling site using GenAlEx 6.503 (Peakall & Smouse, 2006). GenAlEx was also used to estimate population structure with *F*_ST_ *. F*_ST_ estimates were then linearized $\left({\hat{F}}_{\text{ST}}/\left(1-{\hat{F}}_{\text{ST}}\right)\right)$ following the procedure by Rousset (1997). Linearized pairwise ${\hat{F}}_{\text{ST}}s$ were used in all subsequent analyses requiring pairwise ${\hat{F}}_{\text{ST}}s$ input. The level of genetic differentiation among sampling sites was visualized with a principal coordinate analysis (PCoA) of the ${\hat{F}}_{\text{ST}}s$ conducted using GenAlEx 6.503 (Peakall & Smouse, 2006).

Population structure was examined using STRUCTURE 2.2.2 (Pritchard, Stephens, & Donnelly, 2000). The analysis was conducted hierarchically. We first identified clusters examining the entire data set; clusters were then independently subject to further STRUCTURE analyses. This process was continued on individual clusters until no further evidence of population structure was detected. We estimated the most likely number of clusters based on the Evanno methodology (Evanno, Regnaut, & Goudet, 2005) implemented in STRUCTURE HARVESTER v0.6.92 (Earl & vonHoldt, 2012). Each independent STRUCTURE analysis was conducted using five replicate runs, where each run consisted of 2,000,000 iterations with an initial burn‐in of 200,000. The results of these five separate replications were then combined into a single population output using the program CLUMPP 1.1.2 (Jakobsson & Rosenberg, 2007) for the most likely value of *K* (number genetic groups) and visualized using the program DISTRUCT 1.1 (Rosenberg, 2004). A spatial allele autocorrelation analysis was conducted in GenAlEx 6.503 (Peakall & Smouse, 2006). Distance classes (waterway distances) were determined with Google Earth (Google, Mountain view, Chile).

### 2.5 Population differentiation causes

To test whether diversity and differentiation were affected by the number, type (reservoir, run of the river, or natural), and age of the barriers, we conducted a series of Mantel tests (9,999 iterations) in GenAlEx 6.503 (Peakall & Smouse, 2012). We correlated linearized pairwise ${\hat{F}}_{\text{ST}}s\;\left({\hat{F}}_{\text{ST}}/\left(1-{\hat{F}}_{\text{ST}}\right)\right)$ (Rousset, 1997) with: (a) waterway distances (Wwd) between sampling sites; (b) cumulative number of barriers between sampling sites (BaN) pondered by barrier type (see Supporting Information); (c) cumulative age of the barriers (BaA: sum of the years in operation of each barrier between sampling sites); (d) Elevation difference (mean pairwise difference in Elevation between sampling sites); and (e) Slope (mean pairwise Slope between sampling sites calculated according to Stelkens, Jaffuel, Escher, and Wedekind (2012). Decomposed pairwise regression (DPR) after each test was used to identify and remove potential outlier sites, which could be masking the effects of the tested landscape variable (Koizumi, Yamamoto, & Maekawa, 2006; Mccracken, Perry, Keefe, & Ruzzante, 2013). DPR was conducted manually. Sampling sites with 95% confidence intervals not including 0 were considered putative outliers and subsequently removed from the Mantel test until no putative outlier remained. The best model for each test was chosen based on the lowest corrected Akaike's information coefficient (AICc).

We also tested for the effect of distance on connectivity after controlling for the effects of the number (age) of barriers and vice versa with partial Mantel tests. To support the Mantel correlations, we performed a distance‐based redundancy analysis (dbRDA) in R package, using the genetic differentiation matrix (linearized pairwise ${\hat{F}}_{\text{ST}}s\;\left({\hat{F}}_{\text{ST}}/\left(1-{\hat{F}}_{\text{ST}}\right)\right)$ versus Wwd, BaN, BaA, Elevation, Slope, and *A*_R_. This procedure provides options for finding out significant explanatory landscape variables affecting the genetic distances between all sampling sites of the Biobío system, as suggested by Legendre and Anderson (1999).

### 2.6 Anthropogenic and natural fragmentation influences in connectivity

Finally, to understand how anthropogenic and natural fragmentation influences connectivity, we estimated contemporary migration rate among populations with BayesAss 3.0 (Wilson & Rannala, 2003). All BayesAss analyses were conducted using 2,000,000 burn‐in and 20,000,000 iterations. Mixing parameters for migration rate, allele frequencies, and inbreeding coefficients were set at 0.12 0.25, and 0.25, respectively, to achieve acceptance rates within the ideal range of 20% and 60% (Rannala, 2007). Migration estimates with 95% CI that did not include 0 were assumed significant.

We estimated effective population size $\left({\hat{N}}_{e}\right)$ with the linkage disequilibrium method as implemented in LDNe (Waples & Do, 2010) using the *p*_crit_ = .02 as all our sample sizes were >25 (Waples & Do, 2010), with 95% confidence intervals generated via jackknifing between pairs of loci. First, however, potential immigrants were identified with GeneClass2 (Piry et al., 2004) and removed from the data set prior to the estimation of effective population size.

## 3 RESULTS

### 3.1 Genetic quality control

A total of 927 individuals were sequenced and genotyped at a final tally of 28 microsatellite loci after excluding 5 from an initial 33 loci because of missing values in >50% individuals. Also, one marker (Per 50) exhibited signs of null alleles and departures from Hardy–Weinberg in 19 sampling sites; the analyses were performed both with and without this locus, and the results did not change. The remaining 27 loci were free of null alleles and exhibited no LD in any sample site; they also showed no evidence of large allele dropout or scoring errors. Two markers (Per13 and Per 39) were identified as putatively under selection. Diversity indices (*A*_R_, *H*_o_, and *H*_e_) and STRUCTURE results were, however, similar (see Table S1 and Figure S1) whether run with 28 or 26 loci. No genetic structure was detected between sampling years in any of the locations for which data were available for 2016 and 2017 (see Table S2 and Figure S2). All subsequent analyses were carried out with 28 microsatellite loci and 927 individuals. Sample size per sample location and population as well as estimates of *A*_R_, *H*_o_, and *H*_e_ are shown in Table 2. Allelic richness ranged from 5.73 to 9.39. Average heterozygosities over loci were *H*_e_ = 0.643 and *H*_o_ = 0.603 (Table 2). Wright's fixation index varied between 0.006 and 0.098 (Figure S3).

### 3.2 Population structure

**Figure 2:**
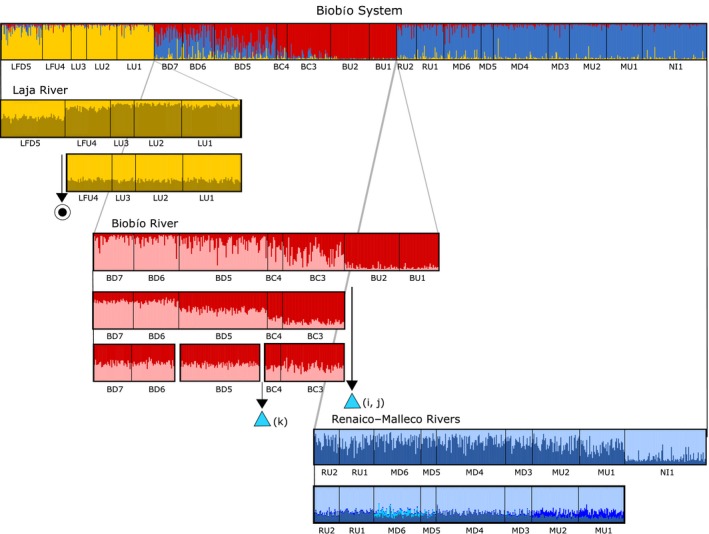
Hierarchical STRUCTURE analysis of the freshwater endemic *Percilia irwini* (using Evanno method) from 21 locations and characterized at 28 microsatellite loci. Vertical color lines indicate individual admixture coefficients (*Q*). Black‐and‐white circle represents the “Salto del Laja” waterfall, (i) Ralco, (j) Pangue, and (k) Angostura hydropower plants

When considering the entire data set, the most likely number of genetic clusters using STRUCTURE was *K* = 3 with clusters corresponding to each of three main rivers comprising the Biobío basin (Laja, Biobío, and Renaico–Malleco rivers, Figure 2; see also Figure S4 for a similar pattern observed with PCoA). Hierarchical analyses considering these three initial clusters separately indicated that the final number of groups was 8. Individuals of the Laja River were pooled into two groups separated by the natural waterfall “Salto del Laja” (Figure 2). Individuals from the Biobío River were pooled into four groups with three of these (BU, BC, and BD) separated by hydroelectrical power plants (Figure 2). The Renaico–Malleco collections grouped into two distinct pools (Figure 2). In the Biobío River, there was a positive autocorrelation (*p* < .001) with distance with neighborhood size = 120 km (Figure S5) indicating an isolation‐by‐distance pattern. A significant autocorrelation was also identified in the Renaico–Malleco River where the neighborhood size was only 30 km. No pattern of autocorrelation was evident for the Laja River.

### 3.3 Causes of population differentiation and barrier influences

#### 3.3.1 Isolation by distance

**Figure 3:**
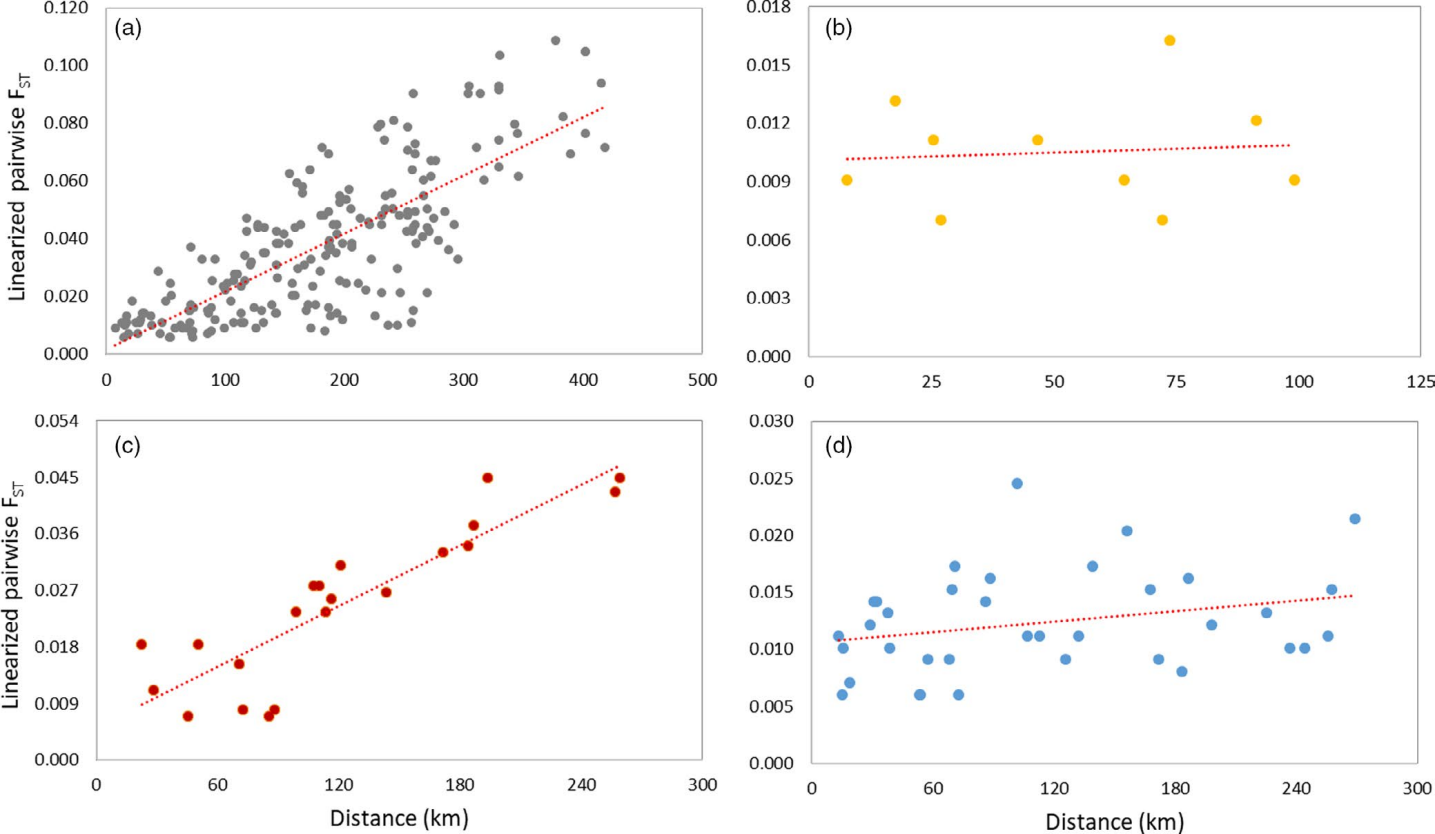
Plots of linearized pairwise ${\hat{F}}_{\text{ST}}s\left({\hat{F}}_{\text{ST}}/\left(1-{\hat{F}}_{\text{ST}}\right)\right)$ estimates versus geographic (waterway) distance (km). The red line indicates the regression line: (a) Biobío system (*R* ^2^ = .5987, *p*‐value* *≤* *.0001), (b) Laja River (*R* ^2^ = .0080, *p*‐value ≥ .384), (c) Biobío River (*R* ^2^ = .7730, *p*‐value* *≤* *.0001), and (d) Renaico–Malleco River (*R* ^2^ = .0798, *p*‐value ≥ .117)

In line with the autocorrelation analysis above, there was evidence of an isolation‐by‐distance (IBD) pattern when *P. irwini* inhabiting all three river systems were considered (*R* ^2^ = .5987, *p* ≤ .0001, Table S3 and Figure S6). The number of barriers (BaN) also had an effect on connectivity when the entire Biobío system was considered (*R* ^2^ = .6751, *p* ≤ .0001); thus, waterway distance and number of barriers both appear to have an effect on genetic differentiation when data from all three rivers are considered. No other variables (age of barrier BaA, Elevation, or Slope) were correlated with genetic differentiation (Table S3) and were thus not considered further. An IBD pattern was also observed within the Biobío River (*R* ^2^ = .7730, *p* ≤ .0001) but not within the Laja (*R* ^2^ = .0080, *p* ≥ .384) or within the Renaico–Malleco River (*R* ^2^ = .0798, *p ≥ *.117; Figure 3, Tables S4–S6). The correlation between the number of barriers (BaN) and genetic distance was significant only for the Biobío River (*R* ^2^ = .6370, *p* ≤ .004) (Table S5).

Given the collinearity between the number of barriers (BaN) and the pairwise waterway distances, two partial Mantel tests were conducted using the information from all locations, first controlling for distance and then testing for the number of barriers, and second, controlling for the number of barriers and then testing for the effect of distance. While waterway distance slightly correlated with genetic distance after controlling for the number of barriers (*R* ^2^ *= *.1907, *p *≤* *.0001), the correlation between the number of barriers and genetic distance was higher and significant (*R* ^2^ *= *.2671, *p *≤* *.0001) after controlling for distance (Table S3).

**Figure 4:**
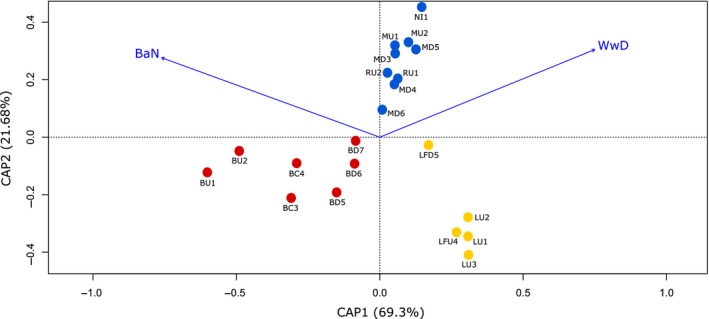
dbRDA plot showing the explanatory landscape variables with significant impact on the (dis)similarities derived from the response (genetic differentiation: linearized ${\hat{F}}_{\text{ST}}s\left({\hat{F}}_{\text{ST}}/\left(1-{\hat{F}}_{\text{ST}}\right)\right)$ values) in the Biobío system: number of barriers (BaN) and waterway distances (Wwd). Yellow points: sampling sites within the Laja River. Red points: sampling sites within Biobío River. Blue points: sampling sites within the Renaico–Malleco River

The distance‐based redundancy analysis (dbRDA) provided results consistent with the Mantel tests. The significant explanatory landscape variables affecting genetic differentiation were waterway distance and number of barriers (Wwd and BaN; *p *≤* *.05; see Table S7 and Figure 4).

#### 3.3.2 Contemporary migration

**Figure 5:**
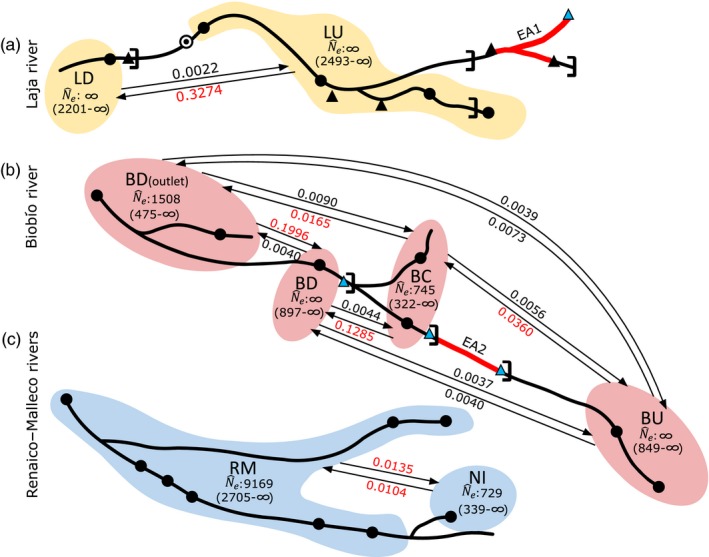
Contemporary migration rates estimated using BAYESASS and effective sizes calculated without migrants in LDNe for each pool of Biobío system: (a) Laja River pool, (b) Biobío River pool, and (c) Renaico–Malleco River pool. Continuous lines show where connectivity is present between populations and their direction. Red contemporary migration rates are significant. Black circles: sample sites. Black‐and‐white circle: “Salto del Laja” waterfall. Black triangles: run‐of‐the‐river hydropower plants. Light blue triangles: storage hydropower plants. Square bracket: physical barrier. Red lines represent areas without *Percilia irwini* individuals (EA: extirpation areas 1 and 2)

In all three rivers, downstream gene flow was generally higher than upstream gene flow (Figure 5). In the Laja River, gene flow was relatively high from LU to the Laja downstream population (LD) and nil in the upstream direction. In the Biobío River, gene flow was similarly significant from Biobío upstream (BU) to Biobío central (BC) and Biobío downstream (BD) but nil or nearly nil in the upstream direction as could be expected from the existence of the three hydropower plants with barriers present in this river (Pangue, Ralco, and Angostura). A lower and symmetrical contemporary gene flow was detected from Niblinto (NI) population to Renaico–Malleco (RM) downstream population (Figure 5).

#### 3.3.3 Effective population sizes $\left({\hat{N}}_{\text{eLD}}\right)$

In general, all populations exhibited a relatively high ${\hat{N}}_{e}$ as suggested by the lower bounds in the confidence intervals (Table 2). The largest effective size was observed in RM (*N*_e_ ~ 9,169, CI: 2,705–∞), while the smallest effective size was detected in BC (*N*_e_ ~ 745, CI: 322–∞). This last population is bound by both upstream and downstream hydroelectric plants. Although data were insufficient for the estimation of effective sizes for some sites (LU, LD, BU, and BD; Figure 5, Table 2), the lower confidence bounds can still be considered as representative of the minimum size of the population.

#### 3.3.4 Evidence of population extirpation

We visited 27 sites overall but were able to collect *P. irwini* from only 21 sites (Figure 1). *Percilia irwini* were absent from two river sections, one in the Laja River (extirpation area 1: EA1, Figure 5) and the other in the Biobío River (extirpation area 2: EA2, Figure 5). Both sections are located between hydroelectric plants. In the Laja River, site EA1 is located between the upstream Abanico (run of the river) and El Toro (Storage hydropower) and the downstream Rucúe (run of the river) plants. In the Biobío River, site EA2 is located between the upstream Ralco and the downstream Pangue plants and both plants have reservoirs and operate by hydropeaking (Figures 1 and 5).

## 4 DISCUSSION

In this study, we have uncovered the influence of both natural and anthropogenic factors on the genetic structure of *P. irwini* inhabiting a spatially complex system that has been subject to varying degrees of human impact. Natural factors including network structure, natural waterfalls, intermittent water flow regimes, and the species' life history characteristics have clearly influenced the genetic structure of *P. irwini* in the two rivers either lacking anthropogenic physical barriers (Renaico–Malleco) or exhibiting waterfall and “run‐of‐the‐river” hydroelectric power plants (Laja). The influence of anthropogenic factors was most apparent in the upper reaches of the Laja River and in the river, most affected by fragmentation, the Biobío River. Below, we discuss the implications of these results considering the species' life history and the type and age of the various anthropogenic barriers to dispersal in existence in these rivers.

### 4.1 Influence of natural factors

At the highest hierarchical level, population structure was detected largely only between rivers with some evidence of gene flow from Renaico–Malleco populations into the lower reaches of the Biobío and of a pattern of IBD when the populations from all three rivers were assessed together (Figures 2 and 4a). Structure within rivers was, however, observed when collections from each river were examined separately: Within the Laja River, *P. irwini* collected downstream of the natural barrier “Salto del Laja” were genetically distinguishable from the upstream aggregations (Figure 2). This waterfall consisting of four falls with a combined altitude difference of >35 m was formed 600 ka BP during episodes of volcanism in the area (Mardones Flores, 2002; Thiele et al., 1998).

There are no barriers to dispersal in the Renaico–Malleco River, and the only variable influencing genetic differentiation in this river was waterway distance (Figure 4). Most samples are genetically indistinguishable from each other except for the most upstream collection along the Malleco River (NI, Figures 2 and 5). Gene flow between this upstream location and all other locations is relatively low but significant in both directions (Figure 5). The Malleco River is subject to a varying rainfall regime with monthly water flow varying between 36 m^3^/s in winter and 2 m^3^/s in summer (Dirección General de Aguas, 2004, 2012). This dependence on rainfall generates intermittency in water flow leading to the disruption of hydrological connectivity and to temporary population isolation (Gasith & Resh, 1999). Water is also extracted from the upper reaches of the Malleco River (i.e., near location NI, Figure 1) for irrigation: Approximately 10,000 ha in the mountain area of this river are irrigated for agriculture, livestock pasture, and forest growth (Dirección General de Aguas, 2004, 2012). We suspect the differentiation between the upstream location and all other ones in this river may at least in part be due to these disruptions in hydrological connectivity caused by the interaction of annual rainfall variation and water diversion in the upper reaches of this river for irrigation.

In the most fragmented river, the Biobío River, gene flow occurs in both directions when no barriers are present: For instance, upstream gene flow appears significant between populations BD and BD_(outlet)_ (Figure 5). Even here though, gene flow is asymmetrical, and the two aggregations are genetically distinguishable. Although there may be various reasons for this genetic differentiation, the fact that BD_(outlet)_ (but not BD) receives migrants from the Renaico–Malleco River (RM) may be a contributing factor.

Although *P. irwini* exhibits low swimming capacity and is unlikely to swim for long periods of time (García et al., 2012), our results provide evidence that *P. irwini* is capable of dispersing in both the upstream and downstream direction in areas unaffected by natural or anthropogenic barriers, but when these are present, dispersal occurs only in the downstream direction. Contemporary migration rates were relatively high in the downstream direction in the lower reaches of the Laja and Biobío rivers, and they were significant and high in both directions between the two groups in the Renaico–Malleco River (Figure 5).

### 4.2 Influence of anthropogenic factors

Aside from the natural “Salto del Laja” barrier, the Laja River also exhibits a total of five run‐of‐the‐river and one storage hydropower plant (which uses the natural Laja Lake as reservoir). Five are located upstream of the “Salto del Laja,” and all are >12 years old while the sixth is located downstream of the natural barrier and was constructed recently (2015). The five upstream hydroelectric power plants, which altogether are responsible for three low‐head dams with lateral intake (as opposed to bottom intake), appear to have had no effect on the genetic structure of *P. irwini* since all collections in this region are genetically indistinguishable forming a single cluster (LU). Hydroelectric power plants with run‐of‐the‐river operation may have lower barrier effects due to their low‐head or mobile dams (Abbasi & Abbasi, 2011; Paish, 2002), yet they are known to affect the immediate physical habitat leading to hydrological impacts such as the reduction of stream width, depth, and current velocity (Anderson, Freeman, & Pringle, 2006; Anderson, Moggridge, Warren, & Shucksmith, 2015; Ovidio, Capra, & Philippart, 2008). Such alterations result in decreasing spawning and rearing grounds and food supply. No *P. irwini* were collected in the stretch of the Laja River located between two sets of power plants (Abanico and El Toro, and Antuco and Rucúe plants) despite considerable sampling effort and historical records that confirm their former presence in those sites (Habit, Belk, & Parra, 2007; Habit, Victoriano, & Parra, 2002). We refer to this stretch of river as extirpation area 1 (EA1, Figure 5). In fact, our results in the upper Laja River are consistent with the suggestion that run‐of‐the‐river dams are responsible for the reduction of fish abundance (Anderson et al., 2006; Jesus, Formigo, Santos, & Tavares, 2004; Ovidio et al., 2008). Also, a translocation experiment conducted in 2001 as a mitigation measure for the interruption of the free displacement of fish in upper Laja River (Habit et al., 2002) involved the transfer of *n* = 852 individuals from near sites LFU4 and LU3 to an upstream area between the Rucúe and the El Toro, Abanico, and Antuco hydroelectric power plants (on the Laja River proper); and to LU1 and LU2 (on the Rucúe River, a tributary to Laja). The absence of differentiation among collections from the upper Laja could in principle be due, at least in part, to this translocation experiment. However, the fact that 31% of all translocated individuals were released in EA1 (Habit et al., 2002) where no *P. irwini* were found is also consistent with the presumed extirpation of this species from this area. This absence or at least severe decline in the abundance of *P. irwini* in EA1 of the Laja River between two sets of long‐established (>38 years, Table 1) hydroelectric power plants suggests a negative impact of these plants on the presence of *P. irwini*. Such negative impact could result from the changes in fluvial geomorphology and flow control following dam construction and operation (Abbasi & Abbasi, 2011; Anderson et al., 2015), as well as from synergistic effects of other human interventions, such as irrigation channels, and invasive salmonids (Habit et al., 2007; Habit, Gonzalez, Ruzzante, & Walde, 2012; Vera‐Escalona, Habit, & Ruzzante, 2019).

The Biobío River is the only river in the drainage with three large dams (>50 m height) that create reservoirs, the effects of which have been described in numerous other systems (Argentina, Angermeier, Hallerman, & Welsh, 2018; Brinker et al., 2018; Dehais, Eudeline, Berrebi, & Argillier, 2010). None of the large dams in the Biobío River exhibit fish passage, ladders, or any other mitigation devices for biological connectivity. The collections within the Biobío were classified into four genetic groups, three of them (BU, BC, and BD) separated by dams. Dams 1 (Ralco) and 2 (Pangue) are >20 years old and operate with hydropeaking. Dam 3 (Angostura), further downstream, began operating in 2014 (Table 1); prior to the dam construction, the river exhibited a canyon likely acting as an old natural barrier. The canyon was flooded with the construction the Angostura power plant. Hydropeaking is known to lead to increases in stranding rates (Nagrodski, Raby, Hasler, Taylor, & Cooke, 2012) and reduced fish abundance (Freeman, Bowen, Bovee, & Irwin, 2001). *Percilia irwini* is highly susceptible to changes in flow regime as a result of a decrease in suitable area with increased water flow (García, Jorde, Habit, Caamaño, & Parra, 2011). Hydropeaking is therefore likely a major driving force affecting the population structure and ultimately the presence of *P. irwini* in the Biobío River. The fact that no *P. irwini* were collected in the section of the Biobío River between dams 1 and 2 is consistent with the hypothesis that the species has been extirpated from this section (EA2, Figure 5). Fluctuations in water level in this section following daily water discharge affect the riparian zone likely preventing successful recruitment. We suspect this is also what happened in the upper reaches of the Laja River as stated above.

Estimates of effective population size were generally high (≈300 to 2,800 when they could be estimated). The effective size for the population in the central section of the Biobío, the section between dams 2 and 3 (BC), was the lowest of all ${\hat{N}}_{e}$ lower limit: 322), likely due to the presence of tandem barriers and a change in passive dispersal. Recent migration rates between BU‐BC and BC‐BD were high in the downstream direction, as also observed in other species (Dehais et al., 2010; Junker et al., 2012). Upstream gene flow was generally nil, as would be expected due to the anthropogenic barriers that prevent upstream migration. Downstream gene flow in the Biobío River despite the numerous dams and their considerable height may be mediated through the passage and survival of *P. irwini* through the turbine system as has been described for other species (Amaral et al., 2015; Dedual, 2007).

### 4.3 Management and conservation comments

Natural barriers and hydroelectric plants with reservoir prevent the movement of *P. irwini* upstream, drastically reducing upstream contemporary gene flow. The size of the population plays an important role in retaining genetic diversity above barriers though, and large effective population sizes may explain why the effects of dams are not yet strongly manifested in the downstream population in Laja and Biobío rivers. The presence of tandem barriers, the consequent change in the characteristics of the physical habitat, and the hydropeaking regimes operating in some of the plants, however, are likely responsible for the two potential instances of population extirpation (EA1 and EA2) and for the drastic reductions in estimated effective sizes (e.g., BC), allelic diversity, and richness (e.g., BC4, Table 2), as has been observed in other studies (Banks et al., 2013). The largest effective size was estimated for the RM population, a population characterized by the absence of insurmountable physical barriers among sampling locations.

Our study demonstrates the importance of studying basin‐wide data sets to understand the strength and direction of anthropogenic impacts on genetic diversity of fish populations. In the upper Laja River, where populations are influenced by run‐of‐the‐river power plants, the maintenance of habitat quality is likely a management priority. In contrast, in the central area of the Biobío River, where the BC population exhibits relatively low effective size, habitat restoration, and increasing connectivity between *P. irwini* populations, using mitigation measures such as fish passes or elevators (Gouskov, Reyes, Wirthner‐Bitterlin, & Vorburger, 2016; Wilkes et al., 2018) is a management priority. Increased research on the life histories of the native fish inhabiting the basin, including on their swimming capacity as well as on the design of fish passes for the maintenance of population connectivity, is needed. Finally, we point out that a new run‐of‐the‐river hydroelectric power plant has been approved in the Renaico–Malleco River (Hydroelectric Agua Viva, Resolución Exenta Nº 1032, 14 September 2017, Región del Biobío) where connectivity is highest. Once in operation, this plant will likely generate great changes in the ecosystem including a decrease in habitat suitability.

Current efforts to increase power generation while reducing greenhouse gas emissions are promoting the development of small hydropower dams as nonconventional renewable energy sources (Habit et al., 2019; Ministerio de Energía, 2016) potentially resulting in increased habitat fragmentation (Díaz et al., 2019), yet ecological and genetic connectivity are either barely considered (ecological) or not considered at all (genetic), in the Chilean environmental impact assessment process (Lacy, Meza, & Marquet, 2017). We suggest the inclusion of genetic analyses as part of an obligatory baseline, which will help monitor the genetic status of populations through genetic variability indicators (e.g., haplotypic diversity, allele richness, gene flow, fixation index, effective population size, among others; Schwartz, Luikart, & Waples, 2007), before, during, and after the life of a project. With such measures in place, it will eventually be possible to discern what changes in genetic diversity are likely to have taken place and then take in situ or ex situ conservation measures necessary to safeguard this diversity in populations at risk.

## CONFLICT OF INTEREST

None declared.

## ETHICAL APPROVAL

The sampling was carried out based on the ethics and biosafety rules and procedures specified in Law 18.755 of the Agricultural and Livestock Service of Chile and the Ethics Committee of Universidad de Concepción.

## Data Availability

Data available from the Dryad Digital Repository: https://doi.org/10.5061/dryad.s1rn8pk3r (Valenzuela‐Aguayo, McCracken, Manosalva, Habit, & Ruzzante, 2019).
