# Supplementary material for: Human‐induced habitat fragmentation effects on connectivity, diversity, and population persistence of an endemic fish, Percilia irwini, in the Biobío River basin (Chile)
Source: Evol Appl. 2019 Dec 9;13(4):794–807. doi: 10.1111/eva.12901 (PMC7086057; doi:10.1111/eva.12901)
Supplement: Supplementary file 1 [file EVA-13-794-s001.docx]

**SUPPLEMENTARY MATERIAL**

Human-induced habitat fragmentation effects on connectivity, diversity and population persistence of an endemic fish, *Percilia irwini,*in the Biobío river basin (Chile)

Valenzuela-Aguayo, Francisca^1,2^, McCracken, Gregory R.^2^, Manosalva, Aliro^1^, Habit, Evelyn^1^ & Daniel E. Ruzzante^2^

**Table S1:** Diversity indexes Allelic richness (*A_R_*), Expected heterozygosity (*He*) and Heterozygosity observed (*Ho*), using 28 or 26 loci microsatellites. N by year (2016, 2017) shows the number of individuals sampled by year.

|  |  |  | **28 loci** | **26 loci** | **28 loci** | **26 loci** | **28 loci** | **26 loci** |
| --- | --- | --- | --- | --- | --- | --- | --- | --- |
| **Site** | **N by year (2016, 2017)** | **N total** | **A_R_** | **A_R_** | **He** | **He** | **Ho** | **Ho** |
| **LU1** | (49, -) | 49 | 5.46 | 5.46 | 0.65 | 0.642 | 0.612 | 0.604 |
| **LU2** | (30, 10) | 40 | 5.36 | 5.39 | 0.645 | 0.643 | 0.61 | 0.609 |
| **LU3** | (20, -) | 20 | 5.32 | 5.33 | 0.626 | 0.620 | 0.62 | 0.612 |
| **LFU4** | (38, -) | 38 | 5.63 | 5.57 | 0.651 | 0.645 | 0.618 | 0.611 |
| **LFD5** | (54, -) | 54 | 6.47 | 6.42 | 0.697 | 0.693 | 0.679 | 0.675 |
| **BU1** | (21, 15) | 36 | 4.51 | 4.69 | 0.55 | 0.579 | 0.539 | 0.566 |
| **BU2** | (35, 16) | 51 | 4.46 | 4.65 | 0.571 | 0.605 | 0.547 | 0.579 |
| **BC3** | (45, 12) | 57 | 5.23 | 5.42 | 0.606 | 0.636 | 0.567 | 0.593 |
| **BC4** | (14, -) | 14 | 4.95 | 5.15 | 0.568 | 0.593 | 0.572 | 0.594 |
| **BD5** | (38, 44) | 82 | 6.13 | 6.27 | 0.654 | 0.673 | 0.623 | 0.642 |
| **BD6** | (42, -) | 42 | 6.45 | 6.53 | 0.673 | 0.679 | 0.617 | 0.628 |
| **BD7** | (37, -) | 37 | 6.4 | 6.47 | 0.66 | 0.670 | 0.625 | 0.640 |
| **RU1** | (22, 14) | 36 | 6.46 | 6.50 | 0.662 | 0.661 | 0.599 | 0.610 |
| **RU2** | (-, 26) | 26 | 6.21 | 6.25 | 0.658 | 0.668 | 0.639 | 0.662 |
| **MU1** | (47, -) | 47 | 6.06 | 6.08 | 0.651 | 0.655 | 0.608 | 0.625 |
| **MU2** | (49, -) | 49 | 6.14 | 6.25 | 0.648 | 0.659 | 0.6 | 0.622 |
| **MD3** | (-, 28) | 28 | 6.13 | 6.17 | 0.639 | 0.647 | 0.601 | 0.619 |
| **MD4** | (32, 40) | 72 | 6.29 | 6.36 | 0.668 | 0.674 | 0.625 | 0.640 |
| **MD5** | (-, 16) | 16 | 6 | 6.01 | 0.639 | 0.649 | 0.593 | 0.607 |
| **MD6** | (49, -) | 49 | 6.42 | 6.44 | 0.667 | 0.676 | 0.591 | 0.604 |
| **NI1** | (55, 29) | 84 | 5.3 | 5.26 | 0.629 | 0.630 | 0.569 | 0.582 |

**
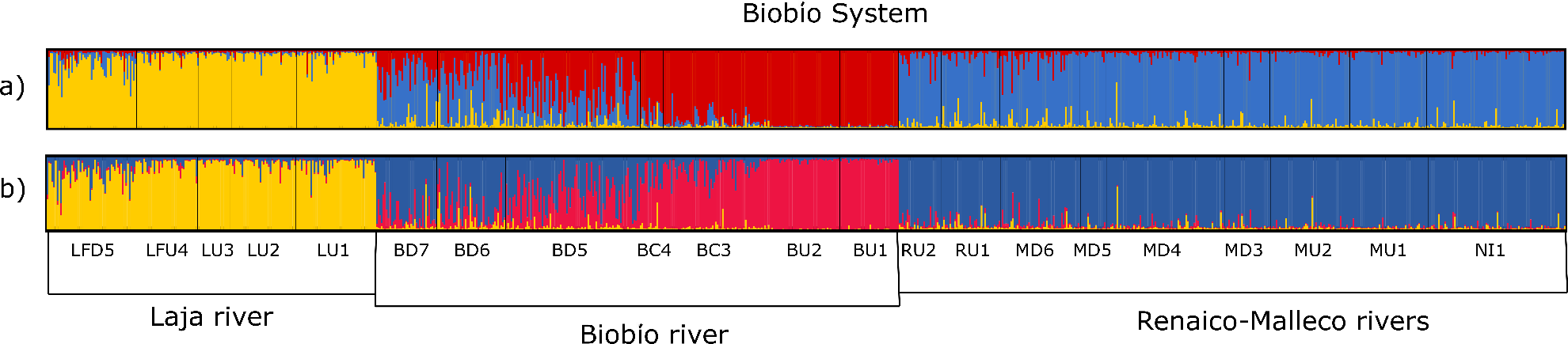
**

**Figure S1:** Hierarchical analysis of freshwater fish *Percilia irwini* from 21 locations and characterized at a) 26 and b) 28 loci. Vertical color lines in the plots show individual admixture coefficients (Q). We estimated the most likely number of clusters based on the Evanno methodology (Evanno, Regnaut, & Goudet, 2005) implemented in STRUCTURE HARVESTER v0.6.92 (Earl & vonHoldt, 2012). Each independent STRUCTURE run was conducted using 5 replicate runs, where each run consisted of 2,000,000 iterations with an initial burn-in of 200,000.

**Table S2:** Pairwise *F_ST_* estimates for the eight sites sampled in 2016 and 2017.

| **2016 Samples** | **LU2** | **BD5** | **BC3** | **BU2** | **BU1** | **RU1** | **MD4** | **NI1** |  |
| --- | --- | --- | --- | --- | --- | --- | --- | --- | --- |
|  | 0 |  |  |  |  |  |  |  | **LU2** |
|  | 0.052 | 0 |  |  |  |  |  |  | **BD5** |
|  | 0.074 | 0.015 | 0 |  |  |  |  |  | **BC3** |
|  | 0.091 | 0.027 | 0.018 | 0 |  |  |  |  | **BU2** |
|  | 0.104 | 0.039 | 0.032 | 0.01 | 0 |  |  |  | **BU1** |
|  | 0.04 | 0.026 | 0.049 | 0.062 | 0.076 | 0 |  |  | **RU1** |
|  | 0.042 | 0.021 | 0.047 | 0.059 | 0.072 | 0.014 | 0 |  | **MD4** |
|  | 0.052 | 0.033 | 0.062 | 0.078 | 0.095 | 0.018 | 0.018 | 0 | **NI1** |
|  | 30 | 38 | 52 | 35 | 21 | 22 | 32 | 55 | **Individuals number** |
| **2017 Samples** | 0 |  |  |  |  |  |  |  | **LU2** |
|  | 0.046 | 0 |  |  |  |  |  |  | **BD5** |
|  | 0.08 | 0.029 | 0 |  |  |  |  |  | **BC3** |
|  | 0.089 | 0.027 | 0.034 | 0 |  |  |  |  | **BU2** |
|  | 0.096 | 0.033 | 0.038 | 0.02 | 0 |  |  |  | **BU1** |
|  | 0.043 | 0.028 | 0.062 | 0.071 | 0.072 | 0 |  |  | **RU1** |
|  | 0.043 | 0.024 | 0.059 | 0.071 | 0.072 | 0.019 | 0 |  | **MD4** |
|  | 0.049 | 0.032 | 0.066 | 0.08 | 0.083 | 0.023 | 0.018 | 0 | **NI1** |
|  | 10 | 44 | 5 | 16 | 15 | 14 | 40 | 29 | **Individuals number** |
| **2016 and** | 0 |  |  |  |  |  |  |  | **LU2** |
| **2017** | 0.046 | 0 |  |  |  |  |  |  | **BD5** |
| **samples** | 0.068 | 0.011 | 0 |  |  |  |  |  | **BC3** |
| **together** | 0.085 | 0.023 | 0.015 | 0 |  |  |  |  | **BU2** |
|  | 0.095 | 0.032 | 0.026 | 0.008 | 0 |  |  |  | **BU1** |
|  | 0.035 | 0.021 | 0.045 | 0.058 | 0.067 | 0 |  |  | **RU1** |
|  | 0.037 | 0.02 | 0.047 | 0.06 | 0.069 | 0.009 | 0 |  | **MD4** |
|  | 0.047 | 0.029 | 0.058 | 0.074 | 0.086 | 0.015 | 0.014 | 0 | **NI1** |
|  | 40 | 82 | 57 | 51 | 36 | 36 | 72 | 84 | **Individuals number** |

**Figure S2:** Correlation between *F_ST_* values obtained for years 2016 and 2017, using 9999 Permutations (R^2^= 0.9358, P-value=0.0001).

**
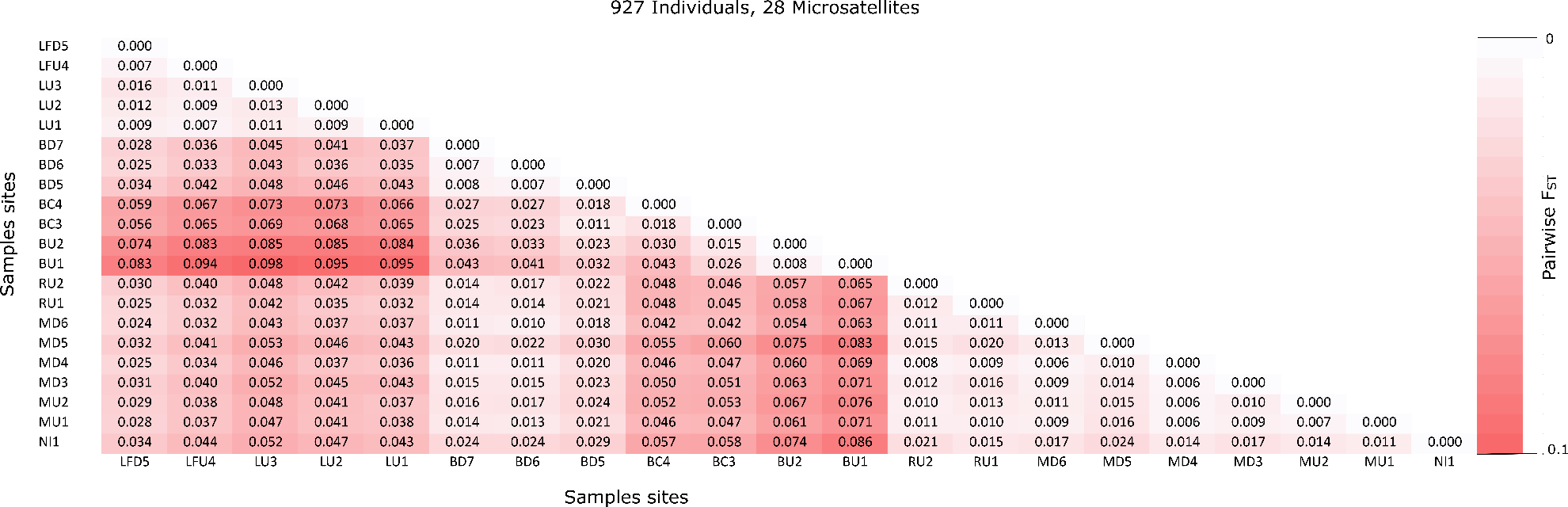
**

**Figure S3**: Heat map depicting pairwise *F_ST_* values calculated using a restricted dataset of 927 individuals collected from twenty-one sampling sites, genotyped at 28 microsatellites.

**
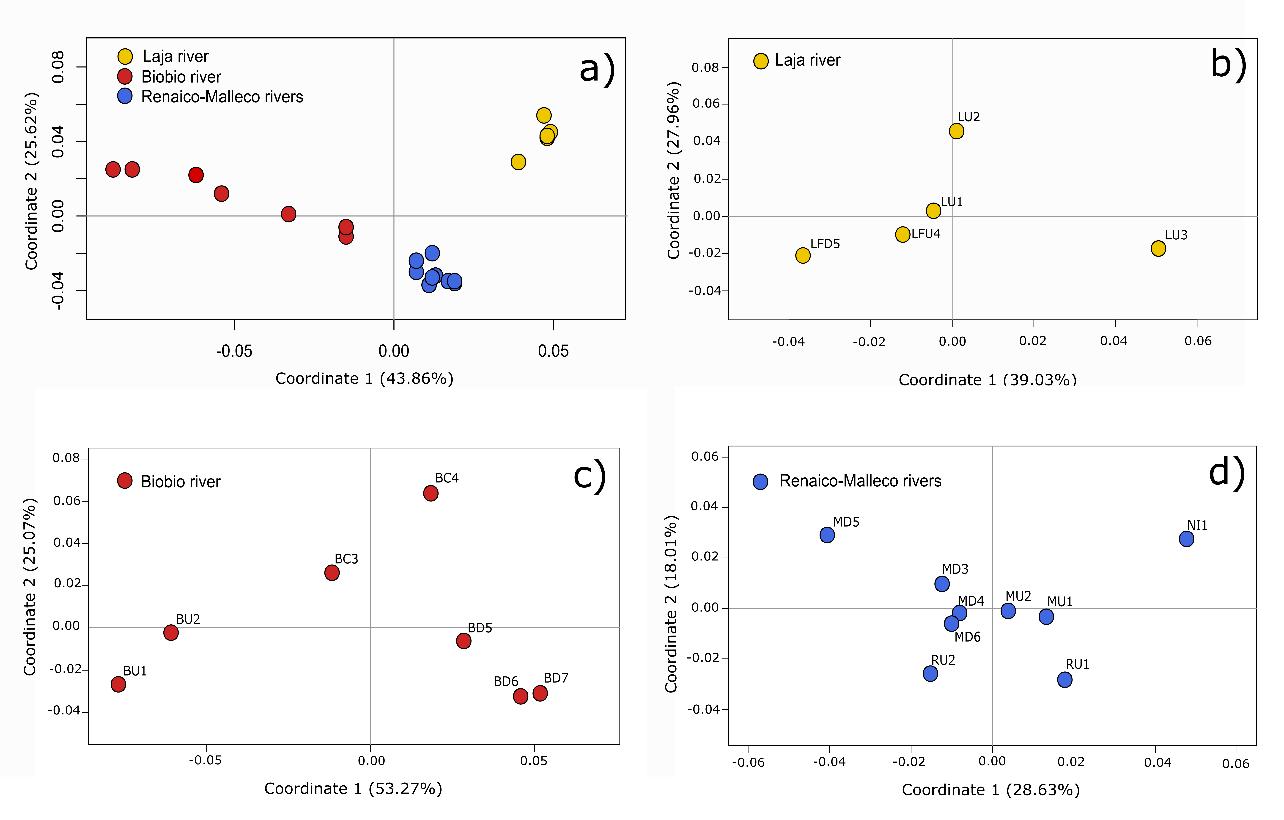
**

**Figure S4:** (a) Principal coordinates analysis based on linearized pairwise $\hat{F}_{ST}$s ($\hat{F}_{ST}$ /(1−$\hat{F}_{ST}$)), a) included all sites of the Biobío system, b) Laja river sites, c) Biobío river sites and d) Renaico-Malleco river sites.


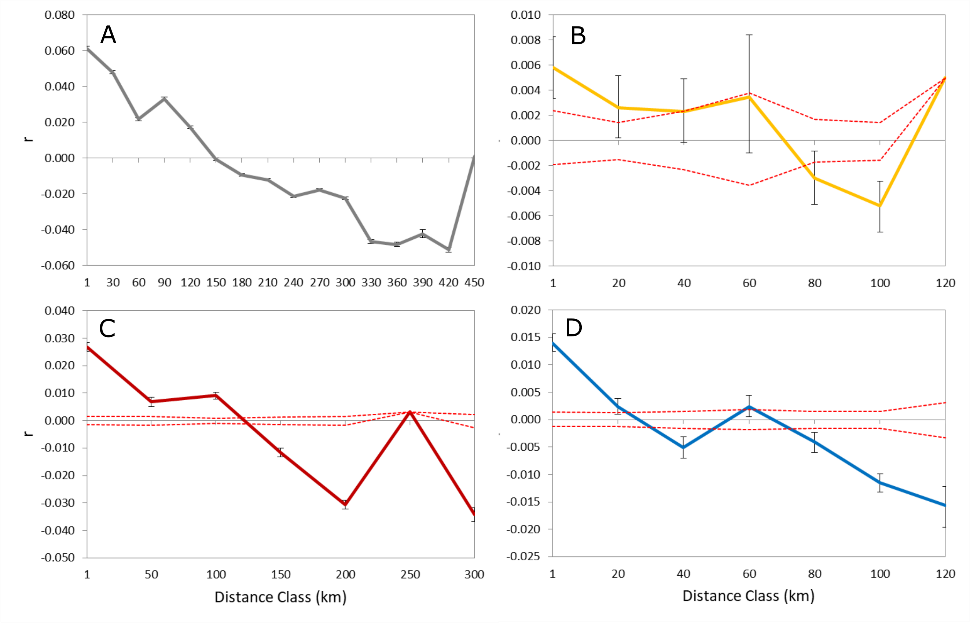


**Table S5:** Spatial allele autocorrelation (r) based in genetic distance plus standard errors (bars) as a function of distance (km) between pairs of individuals: a) Biobío system, b) Laja river, c) Biobío river, d) Renaico-Malleco river.

**Table S3:** Results of Mantel tests between linearized pairwise genetic distances $\hat{F}_{ST}$s ($\hat{F}_{ST}$ /(1−$\hat{F}_{ST}$)) and Waterway distances (Wwd), Number of Barriers (BaN), Age of Barriers (BaA), Elevation and, Slope, considering all sample sites. Potential outlier sites were identified and sequentially removed using a decomposed pairwise regression (Koizumi, Yamamoto, & Maekawa, 2006).

| **ALL BASIN** | **Model** | **Excluded sites** | **R^2^** | **P** | **AICc** |
| --- | --- | --- | --- | --- | --- |
| ${\hat{\mathbf{F}}}_{\mathbf{ST}}$**s vs. Wwd** | 1 | None | 0.5987 | 0.0001 | -137.7405416 |
|  | 2 | RU2 | 0.6454 | 0.0001 | -134.9981760 |
|  | 3 | RU2, RU1 | 0.7416 | 0.0001 | -136.0690234 |
|  | 4 | RU2, RU1, MU1 | 0.7734 | 0.0001 | -133.0741413 |
|  | 5 | RU2, RU1, MU1, NI1 | 0.7939 | 0.0001 | -129.0318904 |
|  | 6 | RU2, RU1, MU1, NI1, MU2 | 0.8239 | 0.0001 | -125.9008207 |
| ${\hat{\mathbf{F}}}_{\mathbf{ST}}$**s vs BaN** | 1 | None | 0.6751 | 0.0001 | -36.57000000 |
| ${\hat{\mathbf{F}}}_{\mathbf{ST}}$**s vs BaA** | 1 | None | 0.1456 | 0.0090 | -27.87184266 |
| ${\hat{\mathbf{F}}}_{\mathbf{ST}}$**s vs Elevation** | 1 | None | 0.0419 | 0.0150 | -26.84091513 |
| ${\hat{\mathbf{F}}}_{\mathbf{ST}}$**s vs. Slope** | 1 | None | 0.0104 | 0.2250 | -26.54983743 |
| **Residual [** ${\hat{\mathbf{F}}}_{\mathbf{ST}}$**s vs BaN] vs Wwd** | 1 | None | 0.1907 | 0.0001 | 38.28947936 |
| **Residual [** ${\hat{\mathbf{F}}}_{\mathbf{ST}}$**s vs Wwd] vs BaN** | 1 | None | 0.2671 | 0.0001 | 48.12041370 |

**a.1**

**a.2**

**b.2**

**Figure S6:** Correlations between pairwise linearized $\hat{F}_{\mathrm{ST}}$s ($\hat{F}_{ST}$ /(1−$\hat{F}_{ST}$)) and a) Waterway distances (Wwd), b) Number of Barriers (BaN), c) Age of Barriers (BaA), d) Elevation and e) Slope, among samples collected from twenty-one sampling sites within the entire Biobío system. 1) Initially all sampling sites were included in the correlation and 2) a barplot of the residuals for each sampling site indicating potential outlier sites.

**Table S4:** Results of Mantel tests between linearized pairwise genetic distances $\hat{F}_{ST}$s ($\hat{F}_{ST}$ /(1−$\hat{F}_{ST}$)) and Waterway distances (Wwd), Number of Barriers (BaN), Age of Barriers (BaA), Elevation and Slope, considering all sample sites of the Laja river system. Potential outlier sites were identified and sequentially removed using a decomposed pairwise regression (Koizumi et al., 2006). Sites exclusions were verified using the best model with the lowest AICc.

| **LAJA RIVER** | **Model** | **Excluded sites** | **R^2^** | **P** | **AICc** |
| --- | --- | --- | --- | --- | --- |
| ${\hat{\mathbf{F}}}_{\mathbf{ST}}$**s vs. Wwd** | 1 | None | 0.0080 | 0.3840 | -90.77985636 |
|  | 2 | Bio07 | 0.0180 | 0.5020 | -90.84387165 |
|  | 3 | Bio07, Bio25 | 0.1850 | 0.6810 | -117.5781737 |
| ${\hat{\mathbf{F}}}_{\mathbf{ST}}$**s vs. BaN** | 1 | None | 0.0110 | 0.4470 | -90.80270008 |
|  | 2 | Bio07 | 0.0140 | 0.4920 | -90.80828619 |
|  | 3 | Bio07, Bio25 | 0.1070 | 0.6770 | -116.7411909 |
| ${\hat{\mathbf{F}}}_{\mathbf{ST}}$**s vs. BaA** | 1 | None | 0.0345 | 0.3490 | -91.00710126 |
|  | 2 | Bio07 | 0.0760 | 0.4990 | -91.33064208 |
|  | 3 | Bio07, Bio25 | 0.2500 | 0.6710 | -118.3100563 |
| ${\hat{\mathbf{F}}}_{\mathbf{ST}}$**s vs. Elevation** | 1 | None | 0.0060 | 0.4570 | -90.76087094 |
|  | 2 | Bio25 | 0.1150 | 0.1710 | -97.70398246 |
|  | 3 | Bio25, Bio22 | 0.4670 | 0.3390 | -128.7705523 |
| ${\hat{\mathbf{F}}}_{\mathbf{ST}}$**s vs. Slope** | 1 | None | 0.1460 | 0.2330 | -92.02572294 |
|  | 2 | Bio07 | 0.6260 | 0.0400 | -98.57667397 |
|  | 3 | Bio07, Bio25 | 0.2780 | 0.3330 | -118.6628457 |

**Table S5:** Results of Mantel tests between linearized pairwise genetic distances $\hat{F}_{\mathrm{ST}}$s ($\hat{F}_{ST}$ /(1−$\hat{F}_{ST}$)) and Waterway distances (Wwd), Number of Barriers (BaN), Age of Barriers (BaA), Elevation and Slope, considering all sample sites of the Biobío river system. Potential outlier sites were identified and sequentially removed using a decomposed pairwise regression (Koizumi et al., 2006).

| **BIOBIO RIVER** | **Model** | **Excluded sites** | **R^2^** | **P** | **AICc** |
| --- | --- | --- | --- | --- | --- |
| ${\hat{\mathbf{F}}}_{\mathbf{ST}}$**s vs. Wwd** | 1 | None | 0.7730 | 0.0001 | -88.34819721 |
|  | 2 | Bio15 | 0.7410 | 0.0030 | -82.71808175 |
|  | 3 | Bio15, Bio21 | 0.7600 | 0.0080 | -79.86766883 |
|  | 4 | Bio15, Bio21, Bio17 | 0.8310 | 0.0450 | -84.60950592 |
|  | 5 | Bio15, Bio21, Bio17, Bio6 | 0.8380 | 0.3340 | -102.0514991 |
| ${\hat{\mathbf{F}}}_{\mathbf{ST}}$**s vs. BaN** | 1 | None | 0.6370 | 0.0040 | -83.72646140 |
|  | 2 | Bio17 | 0.7700 | 0.0160 | -81.35980706 |
|  | 3 | Bio17, Bio15 | 0.7270 | 0.0290 | -77.56633582 |
|  | 4 | Bio17, Bio15, Bio21 | 0.7450 | 0.1240 | -81.30503726 |
|  | 5 | Bio17, Bio15, Bio21, Bio01 | 0.9630 | 0.1630 | -122.4916711 |
| ${\hat{\mathbf{F}}}_{\mathbf{ST}}$**s vs. BaA** | 1 | None | 0.5450 | 0.0010 | -81.53034272 |
|  | 2 | Bio17 | 0.6410 | 0.0170 | -77.33414780 |
|  | 3 | Bio17, Bio15 | 0.5990 | 0.0180 | -74.36933544 |
|  | 4 | Bio17, Bio15, Bio21 | 0.7030 | 0.1690 | -80.08825290 |
|  | 5 | Bio17, Bio15, Bio21, Bio01 | 0.6860 | 0.1670 | -103.1921326 |
| ${\hat{\mathbf{F}}}_{\mathbf{ST}}$**s vs. Elevation** | 1 | None | 0.6701 | 0.0010 | -84.67574557 |
|  | 2 | Bio17 | 0.8709 | 0.0010 | -86.54790259 |
|  | 3 | Bio17, Bio21 | 0.8662 | 0.0090 | -82.61179332 |
|  | 4 | Bio17, Bio21, Bio02 | 0.8732 | 0.0340 | -81.32782988 |
|  | 5 | Bio17, Bio21, Bio02, Bio06 | 0.8067 | 0.1680 | -97.87177964 |
| ${\hat{\mathbf{F}}}_{\mathbf{ST}}$**s vs. Slope** | 1 | None | 0.0125 | 0.3040 | -73.93037854 |
|  | 2 | Bio01 | 0.0320 | 0.2930 | -73.78648009 |
|  | 3 | Bio01, Bio06 | 0.0009 | 0.4600 | -71.37995997 |
|  | 4 | Bio01, Bio06, Bio02 | 0.0159 | 0.5040 | -73.64369273 |
|  | 5 | Bio01, Bio06, Bio02, Bio17 | 0.9894 | 0.3310 | -125.1142184 |
| **Residuals [**${\hat{\mathbf{F}}}_{\mathbf{ST}}$**s vs Wwd] vs BaN** | 1 | None | 0.0880 | 0.1150 | 11.38929464 |
| **Residuals [**${\hat{\mathbf{F}}}_{\mathbf{ST}}$**s vs BaN] vs Wwd** | 1 | None | 0.2209 | 0.0550 | 9.842388483 |

**Table S6:** Results of Mantel tests between linearized pairwise genetic distances $\hat{F}_{\mathrm{ST}}$s ($\hat{F}_{ST}$ /(1−$\hat{F}_{ST}$)) and Waterway distances (Wwd), Elevation and Slope, considering all sample sites of the Renaico-Malleco river system. Potential outlier sites were identified and sequentially removed using a decomposed pairwise regression (Koizumi et al., 2006).

| **RENAICO-MALLECO** | **Model** | **Excluded sites** | **R^2^** | **P** | **AICc** |
| --- | --- | --- | --- | --- | --- |
| ${\hat{\mathbf{F}}}_{\mathbf{ST}}$**s vs. Wwd** | 1 | None | 0.0798 | 0.1170 | -108.6135098 |
|  | 2 | Bio10 | 0.0702 | 0.1760 | -106.0630202 |
|  | 3 | Bio10, Bio26 | 0.2803 | 0.0110 | -107.2197484 |
|  | 4 | Bio10, Bio26, Bio13 | 0.1870 | 0.0400 | -102.9238114 |
|  | 5 | Bio10, Bio26, Bio13, Bio18 | 0.4024 | 0.0530 | -106.0183220 |
|  | 6 | Bio10, Bio26, Bio13, Bio18, Bio11 | 0.1752 | 0.1710 | -107.1794821 |
|  | 7 | Bio10, Bio26, Bio13, Bio18, Bio11, Bio12 | 0.8707 | 0.1610 | -136.3986673 |
| ${\hat{\mathbf{F}}}_{\mathbf{ST}}$**s vs. Elevation** | 1 | None | 0.2289 | 0.0001 | -110.6595875 |
|  | 2 | Bio26 | 0.1387 | 0.0100 | -105.2272072 |
|  | 3 | Bio26, Bio13 | 0.1797 | 0.0280 | -100.5995094 |
|  | 4 | Bio26, Bio13, Bio10 | 0.1587 | 0.0700 | -102.6160528 |
|  | 5 | Bio26, Bio13, Bio10, Bio11 | 0.6417 | 0.0090 | -105.1664580 |
|  | 6 | Bio26, Bio13, Bio10, Bio11, Bio14 | 0.8747 | 0.0360 | -110.1162589 |
|  | 7 | Bio26, Bio13, Bio10, Bio11, Bio14, Bio28 | 0.9741 | 0.1780 | -138.6139097 |
| ${\hat{\mathbf{F}}}_{\mathbf{ST}}$**s vs. Slope** | 1 | None | 0.0062 | 0.3820 | -107.7236500 |
|  | 2 | Bio26 | 0.0053 | 0.3570 | -103.6914510 |
|  | 3 | Bio26, Bio10 | 0.2674 | 0.0540 | -107.0449185 |
|  | 4 | Bio26, Bio10, Bio13 | 0.271 | 0.0820 | -103.9048778 |
|  | 5 | Bio26, Bio10, Bio13, Bio11 | 0.3848 | 0.1490 | -100.6607185 |
|  | 6 | Bio26, Bio10, Bio13, Bio11, Bio27 | 0.3386 | 0.2520 | -109.1578316 |
|  | 7 | Bio26, Bio10, Bio13, Bio11, Bio27, Bio12 | 0.2500 | 0.6630 | -138.0490300 |

**Table S7:** Results of distance-based redundancy analyses conducted to compare genetic distance principal coordinates based on pairwise $\hat{F}_{ST}$s between sampling sites of Biobío basin with significant landscape variables: Waterway distances (Wwd) and Number of Barriers (BaN). Wwd and BaN, were calculated from selecting a number of principal coordinates (PC) between sampling sites. Number of permutations (n), Akaike criterium value (AICc), F-values (F), degrees of freedom (DF), P-values (P) and adjusted R^2^ values are reported. Significant p-values (P)*.

| **Variable** | **N permutation** | **AICc** | **F** | **DF** | **P** | **Adj R^2^** |
| --- | --- | --- | --- | --- | --- | --- |
| **PC1BaN** | 999 | -58.451 | 6.0595 | 1 | 0.002* | 0.20190 |
| **PC1WWd** | 999 | -59.340 | 2.6548 | 1 | 0.002* | 0.26584 |

**LITERATURE CITED**

Earl, D. A., & vonHoldt, B. M. (2012). STRUCTURE HARVESTER: a website and program for visualizing STRUCTURE output and implementing the Evanno method. *Conservation Genetics Resources*, *4*(2), 359–361. https://doi.org/10.1007/s12686-011-9548-7

Evanno, G., Regnaut, S., & Goudet, J. (2005). Detecting the number of clusters of individuals using the software structure: a simulation study. *Molecular Ecology*, *14*(8), 2611–2620. https://doi.org/10.1111/j.1365-294X.2005.02553.x

Koizumi, I., Yamamoto, S., & Maekawa, K. (2006). Decomposed pairwise regression analysis of genetic and geographic distances reveals a metapopulation structure of stream-dwelling Dolly Varden charr. *Molecular Ecology*, *15*(11), 3175–3189. https://doi.org/10.1111/j.1365-294X.2006.03019.x
